# Supervised Classes, Unsupervised Mixing Proportions: Detection of Bots in a Likert-Type Questionnaire

**DOI:** 10.1177/00131644221104220

**Published:** 2022-07-30

**Authors:** Michael John Ilagan, Carl F. Falk

**Affiliations:** 1 McGill University, Montreal, Quebec, Canada

**Keywords:** aberrant responding, bots, Mahalanobis distance, person-total correlation, machine learning

## Abstract

Administering Likert-type questionnaires to online samples risks contamination of the data by malicious computer-generated random responses, also known as bots. Although nonresponsivity indices (NRIs) such as person-total correlations or Mahalanobis distance have shown great promise to detect bots, universal cutoff values are elusive. An initial calibration sample constructed via stratified sampling of bots and humans—real or simulated under a measurement model—has been used to empirically choose cutoffs with a high nominal specificity. However, a high-specificity cutoff is less accurate when the target sample has a high contamination rate. In the present article, we propose the supervised classes, unsupervised mixing proportions (SCUMP) algorithm that chooses a cutoff to maximize accuracy. SCUMP uses a Gaussian mixture model to estimate, unsupervised, the contamination rate in the sample of interest. A simulation study found that, in the absence of model misspecification on the bots, our cutoffs maintained accuracy across varying contamination rates.

In the social sciences, it is common to collect Likert-type self-report data online via crowdsourcing platforms such as Amazon Mechanical Turk (Buhrmester et al., 2011) or Prolific Academic (Peer et al., 2017). Because participants are given monetary compensation, online data collection risks the data being contaminated by survey bots—malicious software submitting invalid data while posing as legitimate human participants. Bot attacks range from crude web-browser-based form fillers to sophisticated server farms (Buchanan & Scofield, 2018; Dennis et al., 2020; Teitcher et al., 2015). As bots are known to inflate Type I and Type II errors as well as bias parameter estimates (Credé, 2010; Huang et al., 2015; Osborne & Blanchard, 2011), it is imperative that researchers detect them in the data.

Bot detection shares common ground with detecting aberrant-responding humans. The common denominator is *content nonresponsivity*, which is that responses are produced irrespective of the item prompt (Meade & Craig, 2012). For humans, content nonresponsivity occurs because they are in a hurry, disinterested, or distracted (DeSimone & Harms, 2018; Meade & Craig, 2012), but for bots, it is because they indiscriminately produce random numbers from some programmed distribution (Buchanan & Scofield, 2018). Although much of the content-nonresponsivity literature is not about bots per se, we regard the methods therein as relevant for “bot detection” insofar as they act on responses being indiscriminately “random” (e.g., Credé, 2010; Hong et al., 2020). To be more precise, a respondent’s *severity* is the proportion of items she answered in a content-nonresponsive manner (Hong et al., 2020) and a bot is presumed to have 100% severity. A focus on bots facilitates plausible statistical assumptions on the nature of “random” responding (Buchanan & Scofield, 2018) that may only sometimes be plausible for human respondents (Niessen et al., 2016).

Human or bot, detection of such aberrant responding is often via nonresponsivity indices (NRIs)—person statistics that, roughly speaking, quantify deviance from the factor or correlational structure of well-behaved participants (Curran, 2016; Meade & Craig, 2012). To name a few, NRIs include classic outlier detection indices (Mahalanobis distance), the similarity of responses to a prototype (person-total correlations), response consistency indices that require multi-item scales or reverse-worded items (even-odd consistency or psychometric antonyms), and model-based statistics (person fit statistics; for example, Dupuis et al., 2019; Meade & Craig, 2012; Niessen et al., 2016).

The premise underlying use of NRIs is that in NRI space, aberrant responders form a different cluster from nonabberant ones (Dupuis et al., 2019, 2020). To illustrate, Figure 1 is a plot of Mahalanobis distance and person-total correlation for a sample of humans and bots, $n_{\mathrm{human}}=50$ and $n_{\mathrm{bot}}=50$, on the Humor Styles Questionnaire (HSQ; Kuiper, 2016). A straight line approximates the boundary between the two classes, human and bot. For each bot, its Likert-type responses were independent draws from a uniform distribution (Dupuis et al., 2019). Deciding whether or not to flag a participant as bot—or in other words, predicting the class of an observation—comes down to finding cutoffs in NRI space (Dupuis et al., 2019; Hong et al., 2020; Huang et al., 2012; Niessen et al., 2016). In Figure 1, the drawn boundary may appear obvious, as the true classes were already labeled, but in a real setting, they are what must be predicted in the first place. The best cutoff, rather than being universal, varies from sample to sample.

**Figure 1.**
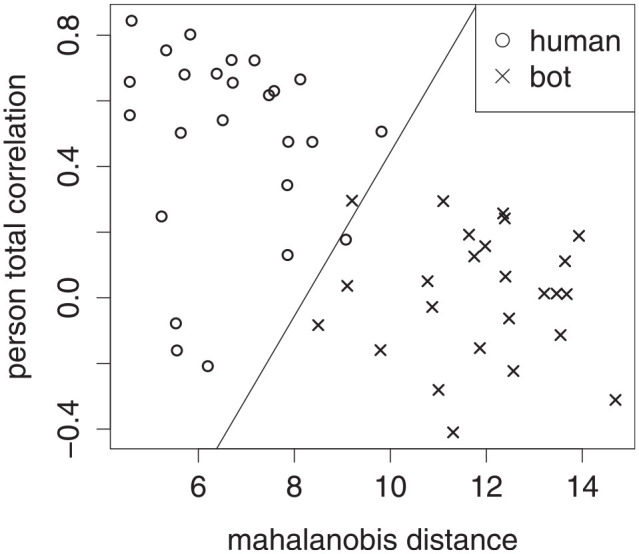
Scatter plot of Mahalanobis Distance Versus Person-Total Correlation for a Sample of 50 Humans and 50 Bots, Where the Bots’ Responses Were From a Uniform Distribution. A Straight Line Approximates the Boundary Between the Two Classes.

Toward detection of content nonresponsivity, the literature on NRIs has typically followed a common program (Dupuis et al., 2019, 2020; Hong et al., 2020; Meade & Craig, 2012; Niessen et al., 2016), which is to compute NRIs on a *calibration sample*—a sample for which the true classes are known. The calibration sample could be a mix of real and simulated data: Well-behaved responses may be actual human responses under interventions to ensure data quality or simulated from some item response model (Dupuis et al., 2019; Hong et al., 2020; Huang et al., 2012; Meade & Craig, 2012; Niessen et al., 2016); aberrant responders may be human participants instructed to respond in a “random” fashion or simulated as such (Huang et al., 2012; Meade & Craig, 2012; Niessen et al., 2016).

The utility of an NRI cutoff is quantified in terms of specificity and sensitivity. In the bot detection context, *specificity* (i.e., the true-negative rate) is the proportion of humans correctly predicted to be human, whereas *sensitivity* (i.e., the true-positive rate) is the proportion of bots correctly predicted to be bots (Hong et al., 2020; Huang et al., 2012; Niessen et al., 2016). The cutoff is then analogous to a critical value in null hypothesis testing—there is a trade-off between specificity and sensitivity, and it is desirable to attain a healthy amount of both.

Although studies on the use of NRIs have shown promise, they fall short of addressing the end goal of NRIs in the first place—the researcher endeavors to, provided her calibration sample, choose an optimal cutoff to predict classes on her target sample. Anecdotally, researchers may choose arbitrary cutoff values based on experience; if done post hoc, this could constitute use of a researcher degree of freedom (Simmons et al., 2011). Some studies go only as far as to show that their chosen NRIs discriminate well between the two classes, not how to find optimal cutoffs (Dupuis et al., 2019, 2020; Meade & Craig, 2012). In Meade and Craig (2012), Mahalanobis distance was shown to be a significant predictor of class in the calibration sample. But significance can be computed only if the class labels were already known, which is not true of the target sample. Other studies (Hong et al., 2020; Niessen et al., 2016) provide recommendations on where to set cutoffs but do so without adequately accounting for base rates – the contamination rate and its complement. In Hong et al. (2020), a parametric bootstrap method was proposed to find cutoffs with 99% nominal specificity, irrespective of the unknown contamination rate. But depending on the contamination rate, a high-specificity cutoff may still end up inaccurate.

To understand the connection between contamination rates and accurate cutoffs, Figure 2 shows a hypothetical NRI where the two classes have the same pair of normal distributions, under two contamination rates. Shifting the cutoff to the left sacrifices specificity in favor of sensitivity, and shifting to the right does the opposite. The most accurate cutoff is where the two density curves cross (James et al., 2013). Clearly, when the contamination rate is low, specificity is more valuable, but when the contamination rate is high, sensitivity is more valuable. For the same pair of normal distributions, Table 1 shows the accuracy of several specificity cutoffs, at different contamination rates. Unfortunately, bots being the majority of the sample is not unheard of (Perkel, 2020), and there is concern over their growing prevalence (Storozuk et al., 2020). We draw attention to the fact that without foreknowledge of the true classes, the density curve in Figure 2B may appear to be a single hump. In that case, an unsuspecting researcher may end up with the false impression that there are few bots in her data, and subsequent use of a cutoff value based on only specificity will prove to be unwise—the result is very low classification accuracy and the sample that remains is mostly bots.

**Figure 2.**
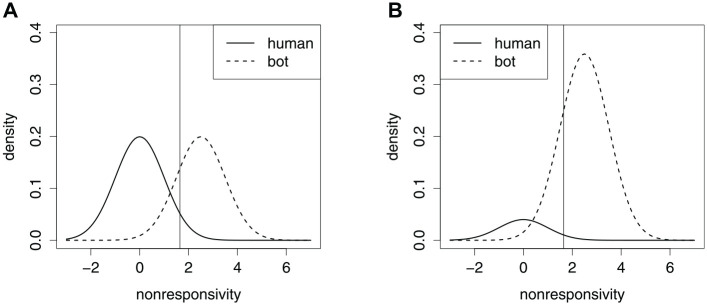
Density Curves for a Hypothetical Nonresponsivity Index, Varying the Contamination Rate. A Vertical Line Locates the Cutoff With 95% Specificity. (A) 50% Contamination. (B) 90% Contamination.

**Table 1.** Accuracy Rates (in Percent) of Specificity (in Percent) Cutoffs Under Different Contamination Rates (in Percent), for the Hypothetical NRI in Figure 2.

Contamination (%)	Accuracy rate (in %) by specificity			
	85% specificity	90% specificity	95% specificity	99% specificity
5	85.4	89.9	94.3	96.9
25	87.0	89.7	91.3	88.5
50	88.9	89.4	87.7	77.9
75	90.9	89.1	84.0	67.4
95	92.4	88.9	81.1	59.0

*Note.* NRI = nonresponsivity index.

The trade-off between specificity and sensitivity is visualized in the receiver operating characteristic (ROC) curve in Figure 3. Every chosen specificity rate (or its complement, false-positive rate) implies a sensitivity rate (i.e., true-positive rate). A larger area under the curve (AUC) is a more favorable trade-off overall, but it shows only the mere fact that it is worth seeking a cutoff, not how to find an accurate one (Unal, 2017). Accuracy is not a property of the NRI as a whole—it is instead a property of a cutoff, provided base rates.

**Figure 3.**
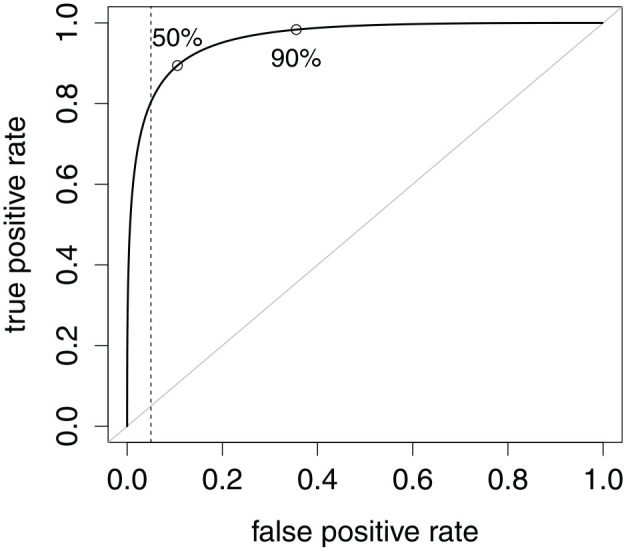
Receiver Operating Characteristic Curve Common to Both Scenarios in Figure 2. Points Locate the Maximum Accuracy Cutoffs Under the Two Contamination Rates. A Vertical Line Locates the Cutoff With 95% Specificity. Area Under the Curve Is 96.1%.

An underappreciated nuance in the NRI literature is that the target sample’s contamination rate cannot be estimated from the calibration sample. For an analogy, suppose a kitchen drawer contained only spoons and forks. Spoon and fork are the classes in the population. If one were to take a simple random sample, the proportion of spoons in that sample would estimate the prevalence of the spoon class. But if one were to take two simple random samples—one sample for spoon and another for fork—the total sample is said to be *stratified* by class (Lohr, 2010). Because the relative sample sizes of spoon and fork are arbitrary in the stratified sample, they cannot estimate the respective prevalences in the drawer population (or any simple random sample thereof). In the bot detection context, calibration samples are stratified by class, so base rates are likewise not available. For example, in Dupuis et al. (2019), a human sample was produced by a pen-and-paper administration of a questionnaire, whereas a bot sample was produced by simulation.

Besides stratified samples, another issue is the incorporation of multiple NRIs into the prediction. The literature typically finds univariate cutoffs, so that each NRI has its own cutoff value (DeSimone & Harms, 2018; Hong & Cheng, 2018; Huang et al., 2012; Niessen et al., 2016). However, doing so ignores the dependence structure between the NRIs. To illustrate, instead of the linear boundary in Figure 1, univariate cutoffs would result in two lines perpendicular to the axes. Furthermore, simultaneous univariate cutoffs amount to multiple testing, which, as in null hypothesis testing, is known to attenuate specificity rate from its nominal level (Bajorski, 2012; Hong et al., 2020).

It is desirable to find accurate multivariate cutoffs empirically, with a stratified calibration sample but without prior knowledge of the contamination rate in the target sample. In the present article, we propose such a method within the machine learning paradigm of supervised learning (James et al., 2013). We build mainly upon Dupuis et al. (2019) and Hong et al. (2020) to highlight underappreciated nuances of the bot detection problem. To be clear, we seek the most accurate cutoff for the data at hand. The remainder of the present article is organized as follows. In the second section, we lay out preliminaries. In the third section, we propose our novel solution, called *Supervised Classes, Unsupervised Mixing Proportions* (SCUMP). In the fourth section, we present a simulation study evaluating the performance of SCUMP under a variety of scenarios. The final section is a discussion.

## Preliminaries

### The Supervised Binary Classification Problem

We start with a d-length vector of Likert-type responses $z_{i}$ for respondent i. We assume that the d items represent ordered categorical data and we later study items with five response options. For completeness, $z_{i}$ can be arranged in a matrix Z where rows are participants and columns are items. Each $z_{i}$ is transformed to a vector of NRIs, $x_{i}$, as done in the literature (Hong et al., 2020; Huang et al., 2012; Meade & Craig, 2012; Niessen et al., 2016). Once NRIs are obtained, we endeavor to transform $x_{i}$ into a prediction of whether respondent i is a bot or human; that is, respondent i’s latent class, $y_{i}$. If the true class labels are known in a calibration sample, this is the supervised binary classification problem in machine learning that we describe next (James et al., 2013).

In the target sample, each observation i=1,…,n has an unknown binary class label $y_{i}\in \{0,1\}$ and a known vector of *features* (or predictors) $x_{i}\in R^{J}$. The data generating process for each independent observation i=1,…,n can be formulated as the hierarchy:

$$\begin{array}{l} y_{i}∼\mathrm{Bernoulli}\left(\lambda \right)\\ x_{i}|y_{i}∼D_{y_{i}}, \end{array}$$

where $D_{0}$ and $D_{1}$ are the feature distributions of the classes. Note that λ is the population prevalence (class prior probability) of class 1. Denote by $n_{0}$ and $n_{1}$ the sample counts of the classes, so that $n=n_{0}+n_{1}$. We consolidate the target sample as

$$X=\left[\begin{array}{c} x_{1}^{T}\\ \vdots \\ x_{n}^{T} \end{array}\right]$$

and $Y=[\begin{array}{cccc} y_{1} & y_{2} & \ldots & y_{n} \end{array}{]}^{T}$.

To make predictions on the unknown Y, we generalize from the calibration sample, called in machine learning the *training set*, whose corresponding feature matrix $X^{\mathrm{tr}}$ and vector of class labels $Y^{\mathrm{tr}}$ are known. We henceforth use the superscript “tr” to denote training set versions of the same quantities. The *classifier* is a function that takes any point in the feature space as input and then outputs its predicted class label. The prediction problem is *supervised* in the sense that there exist data for which ground truth i.e., (the true class labels) is known, to which the classifier’s parameters are fitted. Denote by $\hat{Y}=[\begin{array}{cccc} {\hat{y}}_{1} & {\hat{y}}_{2} & \ldots & {\hat{y}}_{n} \end{array}{]}^{T}$ the vector of predicted classes.

In the bot detection context, we have the vector of class labels Y where we adopt the convention that class 0 is the human class while class 1 is the bot class. Then, $D_{0}$ could be the feature distribution of humans that largely complete items following a confirmatory factor analysis or other item response model (e.g., Hong et al., 2020), whereas $D_{1}$ could be that of the assumption of bot responses being independent uniform variates, as in Dupuis et al. (2019). Participant i=1,…,n is said to be *flagged* if ${\hat{y}}_{i}=1$ and *spared* if ${\hat{y}}_{i}=0$.

If calibration sample bots are generated by the researcher, then the researcher must assume $D_{1}$. The present article focuses on arriving at an accurate classifier given the calibration sample bots—the bots themselves must be instantiated beforehand. Although it is common in the literature to make a uniformity assumption (e.g., Dupuis et al., 2019), there is the possibility of model misspecification—that the calibration $D_{1}$ assumed does not match the true $D_{1}$ in the target sample.

### Nonresponsivity Indices

Common to various NRIs is what we call the *monotone suspicion property*: The NRI may be *suspicion-increasing*, meaning that greater values are more suspicious, or the NRI may be *suspicion-decreasing*, meaning that greater values are less suspicious. In Figure 2, the hypothetical NRI is suspicion-increasing. It is straightforward to recode a suspicion-decreasing NRI into a suspicion-increasing form.

We consider four NRIs whose ROC curves had impressive (e.g., upward of 95%) AUC in Dupuis et al. (2019). Of the four considered NRIs, Mahalanobis distance and person-total correlation are widely used in the literature (Curran, 2016; DeSimone & Harms, 2018; Dupuis et al., 2019, 2020; Meade & Craig, 2012; Niessen et al., 2016). Let z be a respondent vector of raw Likert-type responses (i.e., $z_{i}^{\mathrm{tr}}$ or $z_{i}$).

#### Mahalanobis Distance

The Mahalanobis distance of z is given by

$$D\left(z;m,S\right)=\sqrt{{\left(z-m\right)}^{T}S^{-1}\left(z-m\right)},$$

where m and S are the mean and variance estimated from a reference sample. It is suspicion-increasing and takes nonnegative values.

#### Person-Total Correlation

The person-total correlation of z is given by the sample Pearson correlation between z and *m*, where *m* is a mean vector estimated from a reference sample. It is suspicion-decreasing and ranges from −1 to +1.

Two more NRIs were taken from functional method theory (FMT), *response coherence* and *response reliability* (Dupuis et al., 2015, 2019, 2020). FMT is a testing framework competing with classical test theory and item response theory (Dupuis et al., 2015). Response coherence is suspicion-decreasing, ranging from 0 to 1, and response reliability is suspicion-decreasing, ranging from −1 to +1. Both indices require an assumed number of dimensions that underlie the data. Precise calculation details for FMT-based NRIs are in Supplementary Materials. R code for these FMT-based NRIs was made available in Dupuis et al. (2020).

The four NRIs are outlier statistics, meaning that observations become suspicious by virtue of being outliers relative to some reference sample. In the supervised setting, it makes sense to set the reference sample as the human rows of $Z^{\mathrm{tr}}$. The NRIs then operationalize the dissimilarity of each observation, in both $X^{\mathrm{tr}}$ and X, to the humans of the calibration sample. If the reference sample were badly contaminated, humans may end up outliers (DeSimone & Harms, 2018).

The set of NRIs considered is illustrative rather than exhaustive. There are more NRIs in the literature (e.g., Meade & Craig, 2012). The present article focuses on arriving at an accurate classifier given the features—the features themselves must be chosen beforehand. It is up to the researcher to determine which features they trust to discriminate between human and bot.

### Thresholds and Trade-Offs

For observation i=1,…,n in the target sample, suppose a larger value of $v_{i}\in R$ is more characteristic of class 1, without loss of generality. In the bot detection context, $v_{i}$ may be a single suspicion-increasing NRI or a suspicion-increasing composite of several NRIs. The classifier is given by, for i=1,…,n,

$${\hat{y}}_{i}=\left\{ \begin{array}{cc} 1 & \mathrm{if}\;v_{i}>\;\tau \\ 0 & \mathrm{otherwise}, \end{array}\right.$$

where the threshold τ∈R is chosen to satisfy some criterion in the calibration sample.

For observation i=1,…,n in the target sample, the classification accuracy $p({\hat{y}}_{i}=y_{i})$ is related to specificity $p({\hat{y}}_{i}=0|y_{i}=0)$, and sensitivity $p({\hat{y}}_{i}=1|y_{i}=1)$. Precisely, accuracy is given by

$$p\left({\hat{y}}_{i}=y_{i}\right)=\lambda p\left({\hat{y}}_{i}=1|y_{i}=1\right)+\left(1-\lambda \right)p\left({\hat{y}}_{i}=0|y_{i}=0\right),$$

a weighted mean of specificity and sensitivity, where λ determines the weighting. Then, given $V=[\begin{array}{cccc} v_{1} & v_{2} & \ldots & v_{n} \end{array}{]}^{T}$, choosing τ to satisfy a nominal specificity rate implies an accuracy not guaranteed to be optimal. Calibrating against a high nominal specificity (e.g., 95%) takes for granted that either contamination is low or that the classes are separate enough for it to not matter.

In machine learning, the weighted-mean equation above is typically not directly used. Rather than choosing τ given a known V, maximum likelihood estimation is used to parametrically transform the multivariate $x_{i}$ into a composite feature $v_{i}$, leaving τ fixed. Doing so implicitly maximizes classification accuracy under the assumed parametric model (James et al., 2013). The underlying trade-off between specificity and sensitivity remains relevant, even if they are not modeled directly.

### Machine Learning Classifiers in the Absence of Stratification

To motivate SCUMP, consider a counterfactual where the calibration sample were taken from the hierarchy or mixture rather than stratified. If ground truth were readily available without doing stratified sampling, bot detection would be straightforward, as sample proportions would estimate class prevalences. In particular, consider prediction with logistic regression and Gaussian mixture models, both standard machine learning techniques in supervised classification (James et al., 2013).

In logistic regression, the parameter vector β is estimated by maximum likelihood. Given the parameters, the classifier is given by, for i=1,…,n,

$${\hat{y}}_{i}=\left\{ \begin{array}{cc} 1 & \mathrm{if}\;x_{i}^{T}\beta >0\\ 0 & \mathrm{otherwise}. \end{array}\right.$$

Considering multiple features (e.g., NRIs) simultaneously, the composite is $v_{i}=x_{i}^{T}\beta$ with threshold T = 0 (assuming the intercept is zero or included as a predictor).

More relevant to SCUMP is Gaussian mixture models. Under the assumption that each class’s feature distribution is (multivariate) Gaussian, then the hierarchy can be modeled as (Boos & Stefanski, 2013; James et al., 2013)

$$x_{i}\overset{\mathrm{indep}}{\sim }\lambda N\left(\mu_{1},\Sigma_{1}\right)+\left(1-\lambda \right)N\left(\mu_{0},\Sigma_{0}\right),\;i=1,\ldots ,n.$$

Under such a model, there are two kinds of parameters, estimated separately: the class feature distribution parameters and the mixing proportions.

#### Class Feature Distribution Parameters

For the parameters of $N(\mu_{1},\Sigma_{1})$ and $N(\mu_{0},\Sigma_{0})$, maximum likelihood estimation comes down to simply computing sample means and covariances (without the n−1 correction) by class.

#### Mixture Proportions

For λ and 1−λ, maximum likelihood estimation comes down to sample proportions, $n_{1}^{\mathrm{tr}}/n^{\mathrm{tr}}$ and $n_{0}^{\mathrm{tr}}/n^{\mathrm{tr}}$, for the bot class and the human class, respectively.

Given the parameters, the Bayes classifier (James et al., 2013) is, for i=1,…,n,

$${\hat{y}}_{i}=\left\{ \begin{array}{cc} 1 & \mathrm{if}\;\lambda \phi \left(x_{i};\mu_{1},\Sigma_{1}\right)>\left(1-\lambda \right)\phi \left(x_{i};\mu_{0},\Sigma_{0}\right)\\ 0 & \mathrm{otherwise} \end{array}\right.,$$

where ϕ(x;μ,Σ) is the (multivariate) Gaussian density, with mean μ and variance Σ, evaluated at *x*. Note that the Bayes classifier simply assigns to each observation the class with the higher posterior probability. Considering multiple NRIs, the composite feature is $v_{i}=\lambda \phi (x_{i};\mu_{1},\Sigma_{1})-(1-\lambda )\phi (x_{i};\mu_{0},\Sigma_{0})$ with threshold τ=0.

Note that in both logistic regression and Gaussian mixtures, once parameters are estimated from the training set, the classifier is determined. The prediction on each test set observation depends only on the training set, not on the other test set observations. Also note that in both techniques, the cutoff is multivariate; multiple testing is avoided.

### Classifiers in the Nonresponsivity Literature

With calibration samples stratified, the standard machine learning techniques are not viable. Predictions depend on maximum likelihood estimates, which depend on an arbitrary number of bots, $n_{1}^{\mathrm{tr}}$. In the limit, as the calibration sample proportion of bots approaches 100%, the classifier erroneously learns to flag every respondent irrespective of NRIs.

In a simulation study, Hong et al. (2020) used a human calibration sample of $n_{0}^{\mathrm{tr}}=10,000$ to construct a classifier as follows.

1. For the j th NRI expressed in a suspicion-increasing form, let $\tau^{\left(j\right)}$ be its 99th percentile in the human calibration sample. Thus, the nominal specificity rate is 99%.
2. Flag a respondent if and only if she exceeds the 99th percentile for any (as opposed to all) NRIs. Formally, the classifier is given by, for i=1,…,n,

$${\hat{y}}_{i}=\left\{ \begin{array}{cc} 1 & \mathrm{if}\sum_{j=1}^{J}I\left\{v_{i}^{\left(j\right)}>\tau^{\left(j\right)}\right\}>0\\ 0 & \mathrm{otherwise}, \end{array}\right.$$

where $v_{i}^{\left(j\right)}\in R$ is the j th NRI in suspicion-increasing form and I denotes an indicator of its event.

In the simulation study (Hong et al., 2020), scenarios had contamination rates up to 30%. A multidimensional graded response model was used to simulate human responders in the calibration and target samples. Random uniform responses were simulated for bots in the target sample. Because specificity was the criterion for choosing univariate cutoffs, the calibration sample did not need bots, so $n_{1}^{\mathrm{tr}}=0$. Due to multiple testing, the specificity in the target sample ended up lower (e.g., around 96%) than the nominal 99% rate.

Calibration sample size is an important consideration. Besides the issue of multiple testing, some discrepancy in specificity between calibration and target samples is expected due to sampling error. However, this threat is obscured by the large human calibration size in Hong et al. (2020). Furthermore, having $n_{0}^{\mathrm{tr}}=10,000$ verified humans may be unrealistic.

There is reason to suspect that test length is relevant to the trade-off that a specificity-calibrated classifier would neglect. Intuitively, more items are expected to induce a better AUC: When the inventory has many items, there is a lot of information to distinguish the classes, but for shorter inventories, the gains in specificity translate to dramatic losses in sensitivity. In Hong et al. (2020), both the 27-item test and the 54-item test retained a level of specificity at around 96%, but the sensitivity was around 99% for the longer test versus around 80% for the shorter test. By weighted-mean calculations, such a classifier would have 91.2% accuracy at 30% contamination but 84.8% accuracy at 70% contamination. By the same line of reasoning, the ROCs in Dupuis et al. (2019) may not have been as impressive if they had used a shorter inventory.

Because cutoffs were univariate, besides calibrating for each NRI’s threshold value $\tau^{\left(j\right)}$, Hong et al. (2020) also calibrated for which NRIs to use. For example, it makes sense to exclude Mahalanobis distance from consideration if its inclusion would reduce the classifier’s sensitivity rate. By this logic, a “best subset” of NRIs was identified from their simulation results. Choosing a good subset is important, as univariate cutoffs assume that the NRIs are equally important. In contrast, logistic regression and Gaussian mixtures rely on maximum likelihood to weight features toward maximizing accuracy.

It is possible to have a specificity-calibrated classifier with multivariate cutoffs, without the need to make strong assumptions about the psychometric model for humans as in Hong et al. (2020). One way to do so is to use Mahalanobis distance on the feature space, relative to the human class. Such a classifier is given by, for i=1,…,n,

$${\hat{y}}_{i}=\left\{ \begin{array}{cc} 1 & \mathrm{if}\;D\left(x_{i};\mu_{0},\Sigma_{0}\right)>\tau \\ 0 & \mathrm{otherwise}, \end{array}\right.$$

where $\mu_{0}$ and $\Sigma_{0}$ are the mean and variance estimated from $X^{\mathrm{tr}}$, and τ is empirically chosen to yield the nominal specificity over the calibration sample.

## Proposed Solution

Our proposal assumes that the calibration sample has been obtained in a manner similar to Dupuis et al. (2019)—by policing a sample to ensure that it is human and by generating an arbitrarily large sample of bots from a model assumption. This strategy allows development of cutoff values for the Likert-type questionnaire of interest that may be used in separate samples (future or past) from a similar population. That is, our approach allows predictions about respondent class membership without prior knowledge of contamination rate λ in other samples.

As logistic regression does not explicitly model λ, it makes sense to address the complication induced by stratification in Gaussian mixture models. To be able to predict with a Gaussian mixture model, estimates of the parameters are required. Just as in the nonstratified case, the parameters of the feature distributions $N(\mu_{1},\Sigma_{1})$ and $N(\mu_{0},\Sigma_{0})$ can be estimated supervised, from the calibration sample. But unlike the nonstratified case, it does not make sense to estimate λ from the calibration sample.

Our proposed solution, SCUMP, does estimation in two stages: supervised for the class feature distributions and unsupervised for the mixing proportions.

### Supervised Classes

Estimate class feature distribution parameters $\mu_{0}$, $\Sigma_{0}$, $\mu_{1}$, and $\Sigma_{1}$ supervised on the calibration sample. Precisely, the supervised estimates are given by, for class k=0,1,

$$\left\{ \begin{array}{c} {\hat{\mu }}_{k}=\frac{1}{\sum_{i=1}^{n^{\mathrm{tr}}}I\left\{y_{i}^{\mathrm{tr}}=k\right\}}\sum_{i=1}^{n^{\mathrm{tr}}}I\left\{y_{i}^{\mathrm{tr}}=k\right\}x_{i}^{\mathrm{tr}},\\ {\hat{\Sigma }}_{k}=\frac{1}{\sum_{i=1}^{n^{\mathrm{tr}}}I\left\{y_{i}^{\mathrm{tr}}=k\right\}}\sum_{i=1}^{n^{\mathrm{tr}}}I\left\{y_{i}^{\mathrm{tr}}=k\right\}\left(x_{i}^{\mathrm{tr}}-{\hat{\mu }}_{k}\right){\left(x_{i}^{\mathrm{tr}}-{\hat{\mu }}_{k}\right)}^{T}, \end{array}\right.$$

which are just the Gaussian maximum likelihood estimates within each class.

### Unsupervised Mixing Proportions

Treat the mixture as if the class feature distribution parameters were known to be their supervised estimates. Thus, the model has only one free parameter remaining to estimate, the bot class prior λ, which is obtained unsupervised by maximum likelihood on the target sample. Precisely, the log-likelihood function is given by

$$\ell \left(\lambda \right)=\sum_{i=1}^{n}\log\left(\lambda \phi \left(x_{i};{\hat{\mu }}_{1},{\hat{\Sigma }}_{1}\right)+\left(1-\lambda \right)\phi \left(x_{i};{\hat{\mu }}_{0},{\hat{\Sigma }}_{0}\right)\right),$$

so the unsupervised estimator is given by

$$\hat{\lambda }=\underset{0\leq \lambda \leq 1}{\arg\;\max}\ell \left(\lambda \right).$$

Computationally, we propose a bisection algorithm for finding $\hat{\lambda }$. For ease of notation, for i=1,…,n let

$$\begin{array}{c} \phi_{1}^{\left(i\right)}=\phi \left(x_{i};{\hat{\mu }}_{1},{\hat{\Sigma }}_{1}\right),\\ \phi_{0}^{\left(i\right)}=\phi \left(x_{i};{\hat{\mu }}_{0},{\hat{\Sigma }}_{0}\right). \end{array}$$

Taking the first derivative of the log-likelihood, the maximum likelihood estimator satisfies

$${\left(\frac{d}{d\lambda }\ell \left(\lambda \right)\right)|}_{\lambda =\hat{\lambda }}=\sum_{i=1}^{n}\frac{\phi_{1}^{\left(i\right)}-\phi_{0}^{\left(i\right)}}{\hat{\lambda }\phi_{1}^{\left(i\right)}+\left(1-\hat{\lambda }\right)\phi_{0}^{\left(i\right)}}=0.$$

A global maximum exists, as guaranteed by the second derivative,

$$\frac{d}{d\lambda }\frac{d}{d\lambda }\ell \left(\lambda \right)=\sum_{i=1}^{n}\frac{-{\left(\phi_{1}^{\left(i\right)}-\phi_{0}^{\left(i\right)}\right)}^{2}}{{\left(\lambda \phi_{1}^{\left(i\right)}+\left(1-\lambda \right)\phi_{0}^{\left(i\right)}\right)}^{2}}\leq 0.$$

In R, we plug the derivative of the log-likelihood into the function *stats::uniroot* (R Core Team, 2021) if the endpoints of the search interval {λ∈R:0≤λ≤1} have different signs. Otherwise, $\hat{\lambda }\in \{0,1\}$.

Once all the Gaussian mixture parameters are estimated, we use the Bayes classifier (as defined in the “Preliminaries” section) to make predictions. Note that unlike in the nonstratified case, the parameters estimated supervised are not enough to determine the classifier. Instead, the prediction on each target sample respondent depends not only on the calibration sample but also on the other target sample respondents. In that sense, the classifier is self-adapted to the target sample, even if its contamination rate varies.

**Figure 4.**
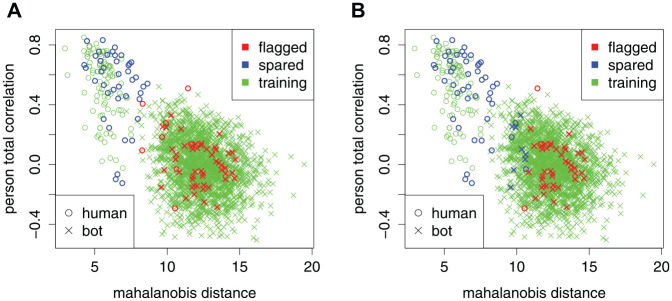
A Demonstration of the SCUMP Bayes Classifier and a Nominal 99% Specificity-Calibrated Classifier on the Same Pair of Calibration and Target Samples. Nonresponsivity Indices Used Were Mahalanobis Distance and Person-Total Correlation. (A) SCUMP Bayes. (B) Nominal 99% Specificity-Calibrated. *Note.* SCUMP = supervised classes, unsupervised mixing proportions.

**Figure 5.**
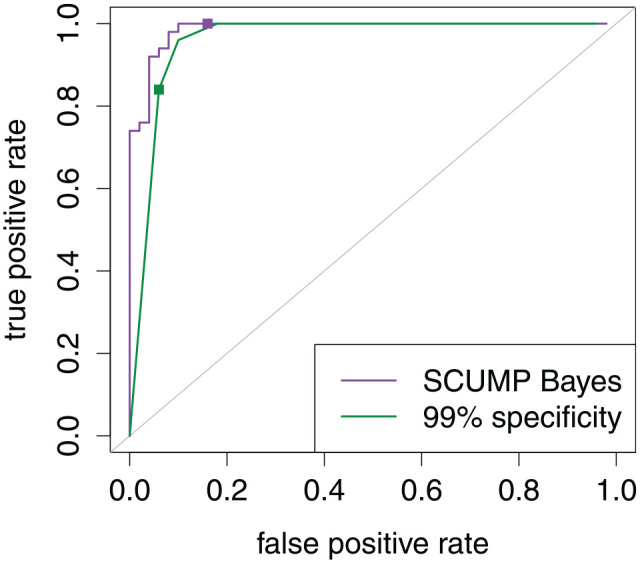
Receiver Operating Characteristic Curves on the Target Sample for the Classifiers Demonstrated in Figure 4. Points Locate the Chosen Cutoffs for the Two Classifiers. Area Under the Curve Was 96.7% for SCUMP Bayes Versus 92.0% for Specificity-Calibrated. *Note.* SCUMP = supervised classes, unsupervised mixing proportions.

We demonstrate the SCUMP Bayes classifier in Figures 4 and 5. Figure 4A shows predictions from SCUMP, using the NRIs Mahalanobis distance and person-total correlation, on the HSQ where the calibration sample had $n_{0}^{\mathrm{tr}}=100$ and $n_{1}^{\mathrm{tr}}=2,000$ and the target sample had $n_{0}=50$ and $n_{1}=50$. Bots were uniform in both the calibration and target samples. The unsupervised estimate for contamination rate is $\hat{\lambda }=0.584$. The confusion matrix (or contingency table) is as follows.

**Table:** 

	flag	spare
bot	50	0
human	8	42

For comparison, Figure 4B shows a nominal 99% specificity-calibrated classifier on the same data. The confusion matrix is as follows.

**Table:** 

	flag	spare
bot	42	8
human	3	47

In this target sample, specificity is 84% for the SCUMP Bayes classifier versus 94% for the specificity-calibrated classifier, but accuracy is 92% for SCUMP Bayes versus 89% for specificity-calibrated classifier. The two ROCs in Figure 5 show that SCUMP improved the predictions in two respects. First, incorporating λ enabled the classifier to lower the specificity rate for better accuracy. In short, the cutoff was chosen adaptively to the target sample. Second, Gaussian mixture posterior probability was simply a better-discriminating composite than the Mahalanobis distance.

Although the researcher could “eyeball” a good-enough nominal specificity rate based on Figure 4, the apparent radius of the bot class depends on $n_{1}^{\mathrm{tr}}$—the more bots in calibration, the farther the radius extends. For example, an observation 10 standard deviations from the mean will be observed eventually if many observations are taken. Furthermore, graphical plots may be difficult to interpret with more than two NRIs, despite the apparent utility of multiple NRIs (DeSimone & Harms, 2018). SCUMP enables the researcher to make $n_{1}^{\mathrm{tr}}$ arbitrarily large and use multiple NRIs without such concerns.

## Simulation Study

### Study Design

To evaluate the performance of the SCUMP Bayes classifier, we conducted a simulation study. For comparison, we also included the specificity-calibrated classifier based on the Mahalanobis distance of the NRIs mentioned earlier in the article. We expected that the specificity-calibrated classifier would incur losses in accuracy at high contamination rates, whereas the SCUMP Bayes classifier would maintain accuracy.

To produce human observations for the simulation study, instead of simulating from a population model, we sampled with replacement from an actual data set. The data set was from a web administration of the HSQ, an inventory of d=32 items, each of which was on a 5-point Likert-type scale. After deleting one row due to having all of its items missing, there were N=1,070 Likert-type rows. The data set was sourced from the Open Psychometrics Project, which did not offer monetary compensation to its participants, so the responses were presumed human.^1^ A single replicate had the following procedure.

#### Generate the Target Sample

To do so, sample $n_{0}$ Likert-type rows from the HSQ data set; then sample $n_{1}$ Likert-type rows from the true bot response distribution. Note that the frequency distribution of the class labels is fixed, rather than allowing random variability drawing from a mixture.

#### Generate the Calibration Sample

To do so, sample $n_{0}^{\mathrm{tr}}$ Likert-type rows from the HSQ data set; then reminiscent of Dupuis et al. (2019), simulate $n_{1}^{\mathrm{tr}}=2,000$ of Likert-type rows as the arbitrarily large sample from a presumed uniform response distribution.

#### Compute NRIs

As a preprocessing step in line with Dupuis et al. (2019), impute all missing values with the middle category, in this case “3.”^2^ Using the calibration sample as reference sample, convert all Likert-type observations to their NRI representations. Use four NRIs: Mahalanobis distance, person-total correlation, response coherence, and response reliability. For the FMT NRIs, assume that there are four factors, which is true of the HSQ (Kuiper, 2016); perform 30 iterations, in line with Dupuis et al. (2020).

#### Make Predictions

From the NRI representations, use the chosen classifier to yield $\hat{Y}$, deciding whether to spare or flag each observation in the target sample.

#### Compute Outcome Measures

Given the ground truth Y, the composite NRI V, and the threshold τ, compute the outcome measures of interest: accuracy, specificity, sensitivity, flag rate, and AUC.

In a 4×2×5×2×2 fully crossed design, the simulation study varied five factors: (a) the number of humans in the calibration sample, $n_{0}^{\mathrm{tr}}\in \{50,100,200,400\}$; (b) the target sample size, n∈{500,1000}; (c) the target sample contamination rate, $n_{1}/(n_{0}+n_{1})\in \{0.05,0.25,0.5,0.75,0.95\}$; (d) the true bot response distribution, $D_{1}$ is either uniform or nonuniform; and (e) the classifier, which was either the nominal 99% specificity-calibrated Mahalanobis distance cutoff or the SCUMP Bayes classifier.

Conditions where the true bot response distribution was nonuniform represented the risks of model misspecification incurred by the uniform bot assumption. The nonuniform response distribution had the probability mass function f such that

$$\left\{ \begin{array}{c} f\left(1\right)=f\left(5\right)=\frac{1}{10},\\ f\left(2\right)=f\left(4\right)=\frac{2}{10},\\ f\left(3\right)=\frac{4}{10}, \end{array}\right.$$

so that bots prefer the middle response category (Simone, 2019).

Each cell had 1,000 replicates. Each outcome measure was averaged over these replicates to yield a Monte Carlo estimate of its cell expected value. Larger values are desired for accuracy, sensitivity, specificity, and AUC. For flag rate, values closer to the contamination rate are desired.

The simulation study was carried out in R version 3.4.0 (R Core Team, 2021) on a desktop computer. The package *mvtnorm* (Genz et al., 2021) was used to compute multivariate Gaussian densities. FMT computations were our own implementation based on Dupuis et al. (2020). For parallel processing, packages *furrr* (Vaughan & Dancho, 2021) and *future* (Bengtsson, 2021) were used. R code to use SCUMP and reproduce this simulation study is publicly available at: https://osf.io/gjmsb/.

### Results

Results were similar across the n=500 and n=1,000 target sample size conditions. For the remainder of the present article, we show only conditions for n=1,000.

**Figure 6.**
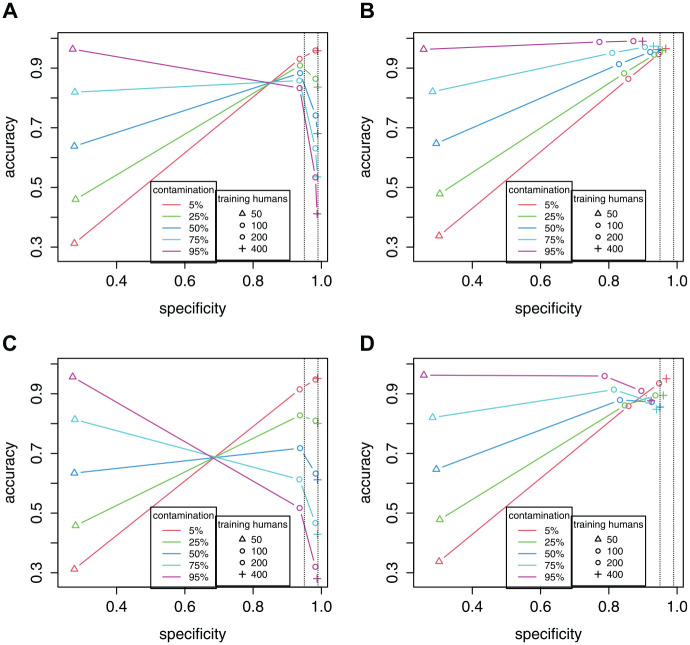
Specificity Versus Accuracy Plots From the Simulation Study. Each Panel Represents a Combination of Classifier and Response Distribution. See Text of Results Section for an Explanation of the Colored Trend Lines. Two Dotted Horizontal Lines Mark 95% and 99% Levels of Specificity. (A) Specificity-Calibrated, Uniform. (B) SCUMP Bayes, Uniform. (C) Specificity-Calibrated, Middle. (D) SCUMP Bayes, Middle. *Note.* SCUMP = supervised classes, unsupervised mixing proportions.

Figure 6 presents the results in specificity versus accuracy plots (see also Supplementary Materials for exact values). Each colored trend line represents a contamination rate (e.g., red is for 5% contamination). Each point represents the number of training humans, increasing from $n_{0}^{\mathrm{tr}}=50$ (plotted as a triangle) to $n_{0}^{\mathrm{tr}}=400$ (plotted as a cross). From such plots, it is easy to see trends in specificity and accuracy as $n_{0}^{\mathrm{tr}}$ increases.

Figure 6A shows the 99% specificity-calibrated classifier under the uniform response distribution. At the lowest $n_{0}^{\mathrm{tr}}=50$, the specificity is well below the nominal rate. Low specificity ends up advantageous at high contamination rates, as many of the bots are correctly flagged. As $n_{0}^{\mathrm{tr}}$ increases, the human class is better estimated, so the specificity rate approaches the nominal 99%. But at the highest $n_{0}^{\mathrm{tr}}$, high specificity ends up very disadvantageous at high contamination rates, as the classifier prioritizes sparing the small minority of humans, letting many bots slip through. Thus, having more training data paradoxically made the classifier worse. The best specificity rate depends on the prevalence of bots, with the trend lines crossing somewhere between $n_{0}^{\mathrm{tr}}=50$ and $n_{0}^{\mathrm{tr}}=100$.

Figure 6B shows the SCUMP Bayes classifier under the uniform response distribution. At the lowest $n_{0}^{\mathrm{tr}}=50$, specificity and accuracy are similar to those of the specificity-calibrated classifier. But as $n_{0}^{\mathrm{tr}}$ increases, both specificity and accuracy increase. The virtue of SCUMP is demonstrated—the classifier adapts specificity to optimize its accuracy, depending on the contamination rate in the target sample. At $n_{0}^{\mathrm{tr}}=100$, SCUMP’s accuracy was no worse than 85% at all contamination rates. At $n_{0}^{\mathrm{tr}}=200$, it was no worse than 94%, and at $n_{0}^{\mathrm{tr}}=400$, it was no worse than 95%.

In Figure 6C and D, we see the effects of nonuniform bots. For the specificity-calibrated classifier in Figure 6C, the trends are of similar form, but the drops in accuracy are sharper. For SCUMP in Figure 6D, the classifier performed well when $n_{0}^{\mathrm{tr}}=100$, but then slightly loses accuracy with more training humans. While SCUMP maintained accuracy at no worse than 80%, as a consequence of misspecification of the bot response distribution it could no longer guarantee that having more training humans would improve the classifier. Finally, in Supplementary Materials we summarize AUCs across bot distribution, classifier, and calibration sample size.

## Discussion

Our simulation results affirm that contamination rate is key to the balancing act between specificity and sensitivity. At 5% contamination, sensitivity had little importance, so the specificity-calibrated classifier had similar accuracy rates to SCUMP Bayes. But at large contamination rates, calibrating for specificity was not the best strategy. In a real setting, the contamination rate is unknown, in which case the choice of nominal specificity (e.g., Hong et al., 2020) is uninformed. SCUMP offers a way out of this conundrum and it was successful relative to the specificity-calibrated classifier.

For specificity-calibrated classifiers, why does specificity end up systematically lower than the nominal? Because the training humans are the reference sample, their observed suspiciousness ends up artificially low. Then, choosing a cutoff with the nominal rate on these low-balled levels of suspicion ends up setting a low bar for suspicion. Consequently, the humans who did not have the privilege of appearing in the calibration sample are flagged at a rate higher than intended. As $n_{0}^{\mathrm{tr}}$ increases, the percentile is better estimated, and the bias vanishes. In the language of machine learning, training error is an underestimate of test error (James et al., 2013; Yarkoni & Westfall, 2017).

Our results extend the 100% severity conditions in Hong et al. (2020) on two aspects. First, we considered contamination rates above the 30% in Hong et al. (2020). Doing so let the trade-off between specificity and sensitivity play out to its logical conclusion—that specificity-calibrated classifiers are unviable at large contamination rates. Second, we considered a smaller number of training humans than in Hong et al. (2020). Doing so demonstrated that in smaller calibration samples, specificity ends up lower than the nominal rate, which may unexpectedly end up benefiting accuracy. Both insights, in our view underappreciated in the literature, benefited from the prediction-oriented perspective of machine learning (Yarkoni & Westfall, 2017).

There are several limitations that we believe can be addressed in future research. Although SCUMP works to fully utilize a stratified calibration sample, it offers no remedy to issues around taking a stratified sample in the first place. SCUMP still relies on assumptions about the bot response distribution (Dupuis et al., 2019). While there is evidence for the existence of bots with uniform response distribution (Buchanan & Scofield, 2018), bots do not all have the same programming (Perkel, 2020). In addition, unlike Hong et al. (2020), we did not study content nonresponsivity in humans, which may have below 100% severity and follow a different process. Without knowing the correct fully-specified population model, detecting a broader spectrum of severity would require a calibration sample free of content nonresponsivity. It is unclear how such a sample could be produced, even with stratification interventions (Huang et al., 2012; Meade & Craig, 2012). We also assumed that human response behavior generalizes from calibration sample to target sample. Depending on how the calibration humans are policed (DeSimone & Harms, 2018; Huang et al., 2012; Meade & Craig, 2012), this generalizability may be questioned. For instance, university samples may be different from online-crowdsourced samples (DeSimone & Harms, 2018; Henrich et al., 2010).

As a future variant of SCUMP, the use of model-based person fit statistics as an NRI may allow portability to other samples that vary in the mean and/or variance of their latent trait distribution. However, the use of person fit usually requires a correctly specified item response model (Meijer & Tendeiro, 2012), that parameter invariance holds, a large human calibration sample ( $n_{0}^{\mathrm{tr}}$ = 250 to 500; de Ayala, 2009), and challenges remain for models with many latent traits. By contrast, we demonstrated that SCUMP with our four chosen NRIs is feasible with a $n_{0}^{\mathrm{tr}}=100$ calibration sample and reaches peak performance around $n_{0}^{\mathrm{tr}}=200$. Although demonstrated with the HSQ (32 items and theoretically four latent dimensions), SCUMP itself does not explicitly require that a measurement model is known for the data and may be more widely applicable. Although other NRIs could be used, the four we chose make few assumptions about the psychometric model for humans or pairing of items with a subscale or latent dimension,^3^ do not require the presence of reverse-worded items, and cannot be easily circumvented by dishonest participants (e.g., bogus items). Nonetheless, we look forward to future innovations with SCUMP.

## Supplemental Material

sj-sty-8-epm-10.1177_00131644221104220 – Supplemental material for Supervised Classes, Unsupervised Mixing Proportions Click here for additional data file. Supplemental material, sj-sty-8-epm-10.1177_00131644221104220 for Supervised Classes, Unsupervised Mixing Proportions by Michael John Ilagan and Carl F. Falk in Educational and Psychological Measurement

sj-tex-1-epm-10.1177_00131644221104220 – Supplemental material for Supervised Classes, Unsupervised Mixing Proportions Click here for additional data file. Supplemental material, sj-tex-1-epm-10.1177_00131644221104220 for Supervised Classes, Unsupervised Mixing Proportions by Michael John Ilagan and Carl F. Falk in Educational and Psychological Measurement

sj-tex-2-epm-10.1177_00131644221104220 – Supplemental material for Supervised Classes, Unsupervised Mixing Proportions Click here for additional data file. Supplemental material, sj-tex-2-epm-10.1177_00131644221104220 for Supervised Classes, Unsupervised Mixing Proportions by Michael John Ilagan and Carl F. Falk in Educational and Psychological Measurement

sj-tex-3-epm-10.1177_00131644221104220 – Supplemental material for Supervised Classes, Unsupervised Mixing Proportions Click here for additional data file. Supplemental material, sj-tex-3-epm-10.1177_00131644221104220 for Supervised Classes, Unsupervised Mixing Proportions by Michael John Ilagan and Carl F. Falk in Educational and Psychological Measurement

sj-tex-4-epm-10.1177_00131644221104220 – Supplemental material for Supervised Classes, Unsupervised Mixing Proportions Click here for additional data file. Supplemental material, sj-tex-4-epm-10.1177_00131644221104220 for Supervised Classes, Unsupervised Mixing Proportions by Michael John Ilagan and Carl F. Falk in Educational and Psychological Measurement

sj-tex-5-epm-10.1177_00131644221104220 – Supplemental material for Supervised Classes, Unsupervised Mixing Proportions Click here for additional data file. Supplemental material, sj-tex-5-epm-10.1177_00131644221104220 for Supervised Classes, Unsupervised Mixing Proportions by Michael John Ilagan and Carl F. Falk in Educational and Psychological Measurement

sj-tex-6-epm-10.1177_00131644221104220 – Supplemental material for Supervised Classes, Unsupervised Mixing Proportions Click here for additional data file. Supplemental material, sj-tex-6-epm-10.1177_00131644221104220 for Supervised Classes, Unsupervised Mixing Proportions by Michael John Ilagan and Carl F. Falk in Educational and Psychological Measurement

sj-tex-7-epm-10.1177_00131644221104220 – Supplemental material for Supervised Classes, Unsupervised Mixing Proportions Click here for additional data file. Supplemental material, sj-tex-7-epm-10.1177_00131644221104220 for Supervised Classes, Unsupervised Mixing Proportions by Michael John Ilagan and Carl F. Falk in Educational and Psychological Measurement

sj-pdf-9-epm-10.1177_00131644221104220 – Supplemental material for Supervised Classes, Unsupervised Mixing Proportions: Detection of Bots in a Likert-Type Questionnaire Click here for additional data file. Supplemental material, sj-pdf-9-epm-10.1177_00131644221104220 for Supervised Classes, Unsupervised Mixing Proportions: Detection of Bots in a Likert-Type Questionnaire by Michael John Ilagan and Carl F. Falk in Educational and Psychological Measurement

## Notes

1.
https://openpsychometrics.org

2. On the prevalence of missing values, 67 rows (6.6%) had exactly one missing item and 10 rows (0.9%) had two or more missing items.

3. Although functional method theory (FMT) approaches require principal component analysis, it does not require a restrictive model as would be required with a between-item multidimensional factor analysis or item response model.
