# Supplementary material for: Supervised Classes, Unsupervised Mixing Proportions: Detection of Bots in a Likert-Type Questionnaire
Source: Educ Psychol Meas. 2022 Jul 30;83(2):217–39. doi: 10.1177/00131644221104220 (PMC9972131; doi:10.1177/00131644221104220)
Supplement: sj-pdf-9-epm-10.1177_00131644221104220 – Supplemental material for Supervised Classes, Unsupervised Mixing Proportions: Detection of Bots in a Likert-Type Questionnaire [file sj-pdf-9-epm-10.1177_00131644221104220.pdf]

Supplementary material for:

Supervised classes, unsupervised mixing proportions: Detection of bots in a Likert-type questionnaire

Michael John Ilagan and Carl F. Falk

Factor space representation in functional method theory

In Functional method theory (FMT), items are represented as unit-length vectors in the factor space (Dupuis et al., 2015). We consider a *matrix of item characteristics*, having d items as rows and k factors as columns. This matrix is defined as satisfying two properties, orthogonal columns and normalized rows.

Orthogonal columns. The k factors are uncorrelated.

Unit-normalized rows. The d items each have Euclidean length 1.

Given a Likert-type response data matrix having n examinees as rows and d items as columns, we want to get the matrix of item characteristics K. To do so, the algorithm is as follows (Dupuis et al., 2020).

1. With the $n \times d$ data matrix as input, calculate the correlation matrix, then do principal component analysis (PCA). Output the $d \times k$ matrix whose columns are the first k eigenvectors.

2. Iteratively satisfy the two properties of the factor space representation until convergence.

   (a) **Orthogonalization.** In the first iteration, use the output of Step 1 as input; otherwise, use the output of Step 2b from the previous iteration. Calculate a correlation matrix, then do another PCA. Output the matrix of $d \times k$ principcal component scores. Note that the output then has uncorrelated columns, but the rows may be unnormalized.

   (b) **Normalization.** With the output of Step 2a, unit-normalize each row. Note that the output then has normalized rows, but the columns may be non-orthogonal. If columns are orthogonal, exit loop.

Figure 7 summarizes the iterative procedure.

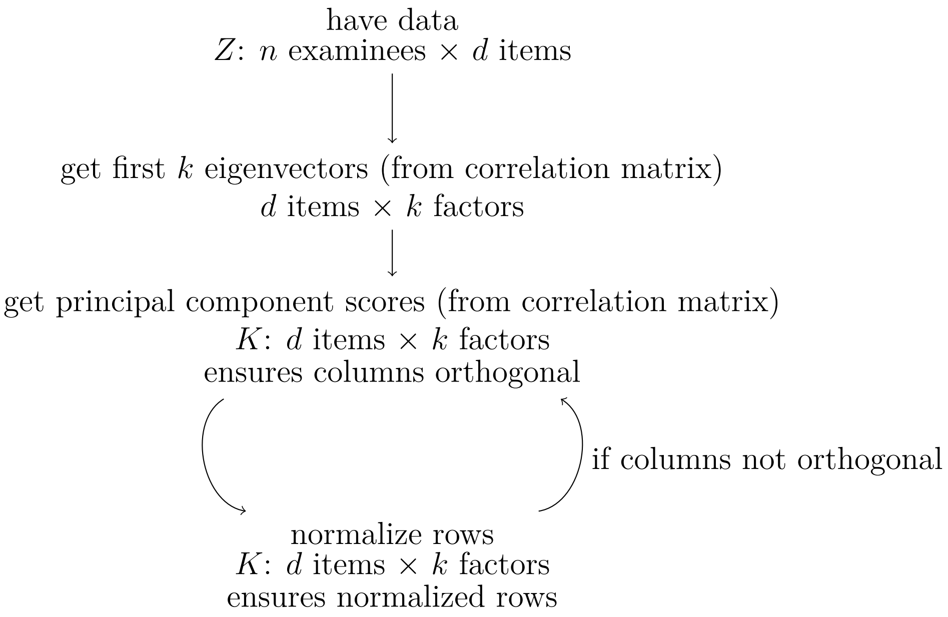

Figure 7. Iterative algorithm for computing the functional method theory (FMT) matrix of item characteristics.

Nonresponsivity indices in factor space

In FMT, examinees are represented in the same factor space as items (Dupuis et al., 2015). We consider an examinee's *response strategy vector*, which is simply a k-dimensional vector collecting the raw response vector's correlations with the factors. Based on response strategy vectors, FMT offers two nonresponsivity indices (Dupuis et al., 2019, 2020): the response coherence; and the response reliability.

Response coherence. The examinee's response coherence is the Euclidean length of her response strategy.

Response reliability. The inner product between the examinee's two unit-normed split-half response strategies.

For response reliability, the split-half procedure is as follows (Dupuis et al., 2020).

1. Construct the matrix of item characteristics for the entire test.

2. Partition into two subtests—subsets of rows—the matrix of item characteristics.

3. Calculate the examinee's two response strategy vectors, one per subset.
4. Unit-norm the two response strategy vectors, take their inner product, then apply Spearman–Brown correction.

Additional Figures and Tables

This section contains one Figure several Tables not present in the main manuscript.

Supplemental Figure 1 is a dot chart of mean-aggregate area under the curve (AUC) in the simulation study (described in the main manuscript). Each dot represents the mean of the AUC aggregated over cells with the same combination of bot distribution, human calibration sample size, and classifier. For example, the topmost dot is the mean AUC over the cells with uniform bots, $n_0^{\text{tr}} = 400$, and Supervised Classes, Unsupervised Mixing Proportions (SCUMP) Bayes classifier. This dot aggregates over different values of contamination rate $\frac{n_1}{n_1+n_0}$, as AUC is invariant to base rates.

Supplemental Tables 1–4 report the results of the simulation study (described in the main manuscript) for the $n = 1000$ conditions. Supplemental Table 1 is for the 99% specificity classifier on uniform bots, corresponding to Figure 6a in the main manuscript; Supplemental Table 2 is for the SCUMP Bayes classifier on uniform bots, corresponding to Figure 6b in the main manuscript; Supplemental Table 3 is for the 99% specificity classifier on middle-responding bots, corresponding to Figure 6c in the main manuscript; and Supplemental Table 4 is for the SCUMP Bayes classifier on middle-responding bots, corresponding to Figure 6d in the main manuscript. Unlike Figure 6 in the main manuscript, these Tables also report sensitivity, flag rate, and AUC.

Supplemental Tables 5–8 is analogous to Supplemental Tables 1–4 but for the $n = 500$ conditions. Unlike Tables 1–4, there are no corresponding Figures in the main manuscript.

Figure 8. Dot chart of mean-aggregate AUCs from the simulation study. Each point aggregates cells under a combination of classifier, bot distribution, and number of calibration humans n_0^{tr}. bayes = Supervised Classes, Unsupervised Mixing Proportions Bayes classifier; spec99 = nominal 99% specificity-calibrated classifier; unif = uniform response style; mrs = middle response distribution; AUC = area under the curve.

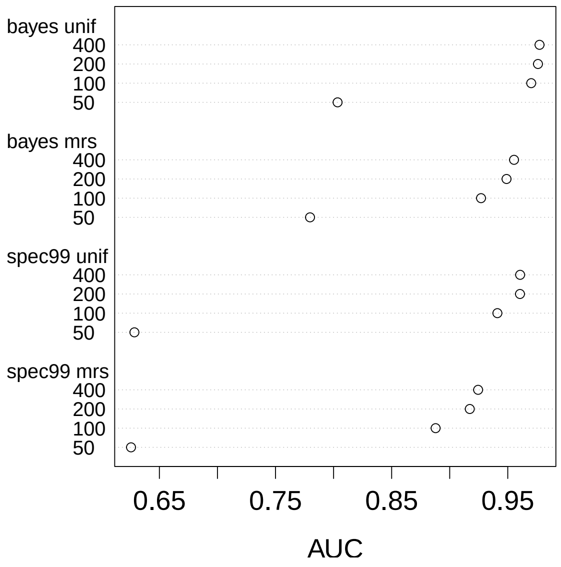

n	contam	n0tr	botdist	classifier	acc	spec	sens	flag	auc
1000	0.05	50	unif	spec99	0.312	0.276	1	0.738	0.636
1000	0.25	50	unif	spec99	0.46	0.28	1	0.79	0.637
1000	0.5	50	unif	spec99	0.638	0.276	1	0.862	0.635
1000	0.75	50	unif	spec99	0.819	0.278	1	0.93	0.634
1000	0.95	50	unif	spec99	0.963	0.271	1	0.986	0.614
1000	0.05	100	unif	spec99	0.931	0.936	0.831	0.102	0.949
1000	0.25	100	unif	spec99	0.909	0.937	0.824	0.253	0.949
1000	0.5	100	unif	spec99	0.883	0.938	0.829	0.446	0.948
1000	0.75	100	unif	spec99	0.858	0.935	0.832	0.64	0.946
1000	0.95	100	unif	spec99	0.833	0.936	0.828	0.789	0.93
1000	0.05	200	unif	spec99	0.959	0.983	0.51	0.042	0.968
1000	0.25	200	unif	spec99	0.864	0.983	0.508	0.14	0.968
1000	0.5	200	unif	spec99	0.741	0.983	0.5	0.258	0.967
1000	0.75	200	unif	spec99	0.631	0.982	0.514	0.39	0.965
1000	0.95	200	unif	spec99	0.534	0.982	0.51	0.485	0.949
1000	0.05	400	unif	spec99	0.958	0.989	0.377	0.03	0.968
1000	0.25	400	unif	spec99	0.836	0.989	0.377	0.103	0.968
1000	0.5	400	unif	spec99	0.681	0.989	0.372	0.192	0.967
1000	0.75	400	unif	spec99	0.535	0.988	0.384	0.291	0.966
1000	0.95	400	unif	spec99	0.411	0.987	0.381	0.363	0.949

Table 2
Simulation study results for $n = 1000$, uniform bots, and 99% specificity classifier. Outcome measures are accuracy, specificity, sensitivity, flag rate, and area under the curve (AUC). n = target sample size, n; contam = contamination rate; n0tr = number of humans in calibration sample, n_0^{tr}; botdist = bot Likert response distribution; acc = accuracy; spec = specificity; sens = sensitivity; flag = flag rate; auc = area under the receiver operating characteristic curve; unif = uniform distribution; mrs = middle responding; scumpbayes = Bayes classifier with Supervised Classes, Unsupervised Mixing Proportions.

n	contam	n0tr	botdist	classifier	acc	spec	sens	flag	auc
1000	0.05	50	unif	scumpbayes	0.338	0.303	1	0.712	0.812
1000	0.25	50	unif	scumpbayes	0.479	0.305	1	0.771	0.816
1000	0.5	50	unif	scumpbayes	0.647	0.294	1	0.853	0.811
1000	0.75	50	unif	scumpbayes	0.821	0.284	1	0.929	0.806
1000	0.95	50	unif	scumpbayes	0.963	0.257	1	0.987	0.785
1000	0.05	100	unif	scumpbayes	0.864	0.857	0.993	0.185	0.978
1000	0.25	100	unif	scumpbayes	0.882	0.845	0.995	0.365	0.978
1000	0.5	100	unif	scumpbayes	0.913	0.83	0.997	0.583	0.977
1000	0.75	100	unif	scumpbayes	0.951	0.81	0.998	0.796	0.975
1000	0.95	100	unif	scumpbayes	0.988	0.772	0.999	0.96	0.959
1000	0.05	200	unif	scumpbayes	0.947	0.946	0.964	0.1	0.983
1000	0.25	200	unif	scumpbayes	0.945	0.933	0.98	0.295	0.983
1000	0.5	200	unif	scumpbayes	0.954	0.921	0.987	0.533	0.983
1000	0.75	200	unif	scumpbayes	0.971	0.906	0.992	0.768	0.981
1000	0.95	200	unif	scumpbayes	0.99	0.872	0.997	0.953	0.965
1000	0.05	400	unif	scumpbayes	0.965	0.967	0.936	0.078	0.985
1000	0.25	400	unif	scumpbayes	0.959	0.956	0.967	0.275	0.985
1000	0.5	400	unif	scumpbayes	0.962	0.945	0.979	0.517	0.984
1000	0.75	400	unif	scumpbayes	0.974	0.931	0.988	0.758	0.982
1000	0.95	400	unif	scumpbayes	0.99	0.899	0.995	0.95	0.966

Table 3

Simulation study results for $n = 1000$, uniform bots, and Supervised Classes, Unsupervised Mixing Proportions (SCUMP) Bayes classifier. Outcome measures are accuracy, specificity, sensitivity, flag rate, and area under the curve (AUC). n = target sample size, n; contam = contamination rate; n0tr = number of humans in calibration sample, n_0^{tr}; botdist = bot Likert response distribution; acc = accuracy; spec = specificity; sens = sensitivity; flag = flag rate; auc = area under the receiver operating characteristic curve; unif = uniform distribution; mrs = middle responding; scumpbayes = Bayes classifier with Supervised Classes, Unsupervised Mixing Proportions.

n	contam	n0tr	botdist	classifier	acc	spec	sens	flag	auc
1000	0.05	50	mrs	spec99	0.312	0.276	0.992	0.737	0.633
1000	0.25	50	mrs	spec99	0.458	0.28	0.992	0.788	0.634
1000	0.5	50	mrs	spec99	0.634	0.276	0.992	0.858	0.632
1000	0.75	50	mrs	spec99	0.814	0.278	0.992	0.925	0.631
1000	0.95	50	mrs	spec99	0.956	0.271	0.993	0.979	0.611
1000	0.05	100	mrs	spec99	0.915	0.936	0.508	0.086	0.896
1000	0.25	100	mrs	spec99	0.828	0.937	0.499	0.172	0.895
1000	0.5	100	mrs	spec99	0.718	0.938	0.498	0.28	0.894
1000	0.75	100	mrs	spec99	0.613	0.935	0.505	0.395	0.893
1000	0.95	100	mrs	spec99	0.517	0.936	0.495	0.473	0.876
1000	0.05	200	mrs	spec99	0.948	0.983	0.286	0.031	0.924
1000	0.25	200	mrs	spec99	0.81	0.983	0.291	0.086	0.925
1000	0.5	200	mrs	spec99	0.632	0.983	0.282	0.149	0.923
1000	0.75	200	mrs	spec99	0.467	0.982	0.295	0.225	0.922
1000	0.95	200	mrs	spec99	0.32	0.982	0.285	0.272	0.907
1000	0.05	400	mrs	spec99	0.951	0.989	0.24	0.023	0.932
1000	0.25	400	mrs	spec99	0.801	0.989	0.236	0.067	0.932
1000	0.5	400	mrs	spec99	0.612	0.989	0.235	0.123	0.93
1000	0.75	400	mrs	spec99	0.429	0.988	0.243	0.185	0.929
1000	0.95	400	mrs	spec99	0.28	0.987	0.243	0.232	0.914

Table 4

Simulation study results for $n = 1000$, non-uniform bots, and 99% specificity classifier. Outcome measures are accuracy, specificity, sensitivity, flag rate, and area under the curve (AUC). n = target sample size, n; contam = contamination rate; n0tr = number of humans in calibration sample, n_0^{tr}; botdist = bot Likert response distribution; acc = accuracy; spec = specificity; sens = sensitivity; flag = flag rate; auc = area under the receiver operating characteristic curve; unif = uniform distribution; mrs = middle responding; scumpbayes = Bayes classifier with Supervised Classes, Unsupervised Mixing Proportions.

n	contam	n0tr	botdist	classifier	acc	spec	sens	flag	auc
1000	0.05	50	mrs	scumpbayes	0.337	0.303	0.999	0.712	0.788
1000	0.25	50	mrs	scumpbayes	0.478	0.305	0.999	0.771	0.791
1000	0.5	50	mrs	scumpbayes	0.647	0.295	0.999	0.852	0.787
1000	0.75	50	mrs	scumpbayes	0.82	0.284	0.999	0.928	0.783
1000	0.95	50	mrs	scumpbayes	0.962	0.258	1	0.987	0.763
1000	0.05	100	mrs	scumpbayes	0.859	0.858	0.881	0.179	0.934
1000	0.25	100	mrs	scumpbayes	0.861	0.847	0.904	0.341	0.934
1000	0.5	100	mrs	scumpbayes	0.879	0.833	0.925	0.546	0.933
1000	0.75	100	mrs	scumpbayes	0.914	0.815	0.947	0.757	0.932
1000	0.95	100	mrs	scumpbayes	0.96	0.788	0.969	0.931	0.916
1000	0.05	200	mrs	scumpbayes	0.935	0.947	0.703	0.085	0.956
1000	0.25	200	mrs	scumpbayes	0.895	0.936	0.769	0.24	0.956
1000	0.5	200	mrs	scumpbayes	0.872	0.926	0.819	0.447	0.955
1000	0.75	200	mrs	scumpbayes	0.877	0.913	0.865	0.671	0.954
1000	0.95	200	mrs	scumpbayes	0.91	0.895	0.91	0.87	0.938
1000	0.05	400	mrs	scumpbayes	0.951	0.968	0.614	0.061	0.963
1000	0.25	400	mrs	scumpbayes	0.895	0.96	0.699	0.205	0.963
1000	0.5	400	mrs	scumpbayes	0.855	0.95	0.761	0.406	0.962
1000	0.75	400	mrs	scumpbayes	0.848	0.94	0.817	0.628	0.96
1000	0.95	400	mrs	scumpbayes	0.877	0.924	0.874	0.834	0.944

Table 5

Simulation study results for $n = 1000$, non-uniform bots, and Supervised Classes, Unsupervised Mixing Proportions (SCUMP) Bayes classifier. Outcome measures are accuracy, specificity, sensitivity, flag rate, and area under the curve (AUC). n = target sample size, n; contam = contamination rate; n0tr = number of humans in calibration sample, n_0^{tr}; botdist = bot Likert response distribution; acc = accuracy; spec = specificity; sens = sensitivity; flag = flag rate; auc = area under the receiver operating characteristic curve; unif = uniform distribution; mrs = middle responding; scumpbayes = Bayes classifier with Supervised Classes, Unsupervised Mixing Proportions.

n	contam	n0tr	botdist	classifier	acc	spec	sens	flag	auc
500	0.05	50	unif	spec99	0.311	0.274	1	0.739	0.634
500	0.25	50	unif	spec99	0.458	0.277	1	0.792	0.635
500	0.5	50	unif	spec99	0.638	0.277	1	0.861	0.633
500	0.75	50	unif	spec99	0.82	0.282	1	0.929	0.632
500	0.95	50	unif	spec99	0.964	0.28	1	0.986	0.595
500	0.05	100	unif	spec99	0.931	0.937	0.83	0.102	0.948
500	0.25	100	unif	spec99	0.908	0.935	0.827	0.255	0.946
500	0.5	100	unif	spec99	0.884	0.936	0.832	0.448	0.946
500	0.75	100	unif	spec99	0.863	0.933	0.84	0.647	0.941
500	0.95	100	unif	spec99	0.829	0.936	0.823	0.785	0.909
500	0.05	200	unif	spec99	0.959	0.983	0.516	0.042	0.967
500	0.25	200	unif	spec99	0.867	0.982	0.523	0.144	0.966
500	0.5	200	unif	spec99	0.745	0.983	0.506	0.262	0.965
500	0.75	200	unif	spec99	0.63	0.982	0.512	0.389	0.961
500	0.95	200	unif	spec99	0.526	0.983	0.502	0.478	0.928
500	0.05	400	unif	spec99	0.958	0.988	0.376	0.03	0.967
500	0.25	400	unif	spec99	0.838	0.988	0.386	0.105	0.967
500	0.5	400	unif	spec99	0.686	0.988	0.384	0.198	0.965
500	0.75	400	unif	spec99	0.528	0.989	0.374	0.283	0.961
500	0.95	400	unif	spec99	0.41	0.988	0.38	0.362	0.929

Table 6

Simulation study results for $n = 500$, uniform bots, and 99% specificity classifier. Outcome measures are accuracy, specificity, sensitivity, flag rate, and area under the curve (AUC). n = target sample size, n; contam = contamination rate; n0tr = number of humans in calibration sample, n_0^{tr}; botdist = bot Likert response distribution; acc = accuracy; spec = specificity; sens = sensitivity; flag = flag rate; auc = area under the receiver operating characteristic curve; unif = uniform distribution; mrs = middle responding; scumpbayes = Bayes classifier with Supervised Classes, Unsupervised Mixing Proportions.

n	contam	n0tr	botdist	classifier	acc	spec	sens	flag	auc
500	0.05	50	unif	scumpbayes	0.341	0.306	1	0.709	0.816
500	0.25	50	unif	scumpbayes	0.474	0.299	1	0.776	0.81
500	0.5	50	unif	scumpbayes	0.647	0.295	1	0.853	0.809
500	0.75	50	unif	scumpbayes	0.822	0.287	1	0.928	0.805
500	0.95	50	unif	scumpbayes	0.963	0.258	1	0.987	0.764
500	0.05	100	unif	scumpbayes	0.866	0.859	0.993	0.183	0.977
500	0.25	100	unif	scumpbayes	0.881	0.843	0.995	0.367	0.976
500	0.5	100	unif	scumpbayes	0.914	0.832	0.997	0.583	0.975
500	0.75	100	unif	scumpbayes	0.951	0.81	0.998	0.796	0.97
500	0.95	100	unif	scumpbayes	0.988	0.775	0.999	0.96	0.938
500	0.05	200	unif	scumpbayes	0.948	0.947	0.966	0.099	0.982
500	0.25	200	unif	scumpbayes	0.945	0.933	0.98	0.295	0.982
500	0.5	200	unif	scumpbayes	0.954	0.922	0.987	0.533	0.98
500	0.75	200	unif	scumpbayes	0.97	0.905	0.992	0.768	0.976
500	0.95	200	unif	scumpbayes	0.99	0.87	0.997	0.953	0.944
500	0.05	400	unif	scumpbayes	0.966	0.967	0.937	0.078	0.984
500	0.25	400	unif	scumpbayes	0.959	0.956	0.967	0.275	0.983
500	0.5	400	unif	scumpbayes	0.962	0.945	0.979	0.517	0.982
500	0.75	400	unif	scumpbayes	0.974	0.931	0.988	0.758	0.978
500	0.95	400	unif	scumpbayes	0.99	0.899	0.995	0.95	0.945

Table 7

Simulation study results for $n = 500$, uniform bots, and Supervised Classes, Unsupervised Mixing Proportions (SCUMP) Bayes classifier. Outcome measures are accuracy, specificity, sensitivity, flag rate, and area under the curve (AUC). n = target sample size, n; contam = contamination rate; n0tr = number of humans in calibration sample, n_0^{tr}; botdist = bot Likert response distribution; acc = accuracy; spec = specificity; sens = sensitivity; flag = flag rate; auc = area under the receiver operating characteristic curve; unif = uniform distribution; mrs = middle responding; scumpbayes = Bayes classifier with Supervised Classes, Unsupervised Mixing Proportions.

n	contam	n0tr	botdist	classifier	acc	spec	sens	flag	auc
500	0.05	50	mrs	spec99	0.31	0.274	0.992	0.739	0.631
500	0.25	50	mrs	spec99	0.456	0.277	0.992	0.79	0.632
500	0.5	50	mrs	spec99	0.635	0.277	0.992	0.858	0.63
500	0.75	50	mrs	spec99	0.815	0.282	0.992	0.923	0.629
500	0.95	50	mrs	spec99	0.956	0.28	0.991	0.978	0.594
500	0.05	100	mrs	spec99	0.915	0.937	0.501	0.085	0.895
500	0.25	100	mrs	spec99	0.828	0.935	0.505	0.175	0.893
500	0.5	100	mrs	spec99	0.721	0.936	0.507	0.285	0.893
500	0.75	100	mrs	spec99	0.621	0.933	0.517	0.405	0.887
500	0.95	100	mrs	spec99	0.519	0.936	0.497	0.475	0.857
500	0.05	200	mrs	spec99	0.948	0.983	0.295	0.031	0.923
500	0.25	200	mrs	spec99	0.811	0.982	0.297	0.088	0.923
500	0.5	200	mrs	spec99	0.637	0.983	0.29	0.154	0.922
500	0.75	200	mrs	spec99	0.461	0.982	0.287	0.22	0.917
500	0.95	200	mrs	spec99	0.322	0.983	0.288	0.274	0.887
500	0.05	400	mrs	spec99	0.951	0.988	0.235	0.023	0.931
500	0.25	400	mrs	spec99	0.803	0.988	0.246	0.07	0.93
500	0.5	400	mrs	spec99	0.616	0.988	0.243	0.128	0.928
500	0.75	400	mrs	spec99	0.418	0.989	0.227	0.173	0.924
500	0.95	400	mrs	spec99	0.281	0.988	0.244	0.233	0.896

Table 8

Simulation study results for $n = 500$, non-uniform bots, and 99% specificity classifier. Outcome measures are accuracy, specificity, sensitivity, flag rate, and area under the curve (AUC). n = target sample size, n; contam = contamination rate; n0tr = number of humans in calibration sample, n_0^{tr}; botdist = bot Likert response distribution; acc = accuracy; spec = specificity; sens = sensitivity; flag = flag rate; auc = area under the receiver operating characteristic curve; unif = uniform distribution; mrs = middle responding; scumpbayes = Bayes classifier with Supervised Classes, Unsupervised Mixing Proportions.

n	contam	n0tr	botdist	classifier	acc	spec	sens	flag	auc
500	0.05	50	mrs	scumpbayes	0.341	0.306	0.999	0.709	0.79
500	0.25	50	mrs	scumpbayes	0.474	0.299	0.999	0.776	0.786
500	0.5	50	mrs	scumpbayes	0.647	0.295	0.999	0.852	0.785
500	0.75	50	mrs	scumpbayes	0.821	0.287	0.999	0.927	0.781
500	0.95	50	mrs	scumpbayes	0.962	0.259	1	0.987	0.743
500	0.05	100	mrs	scumpbayes	0.861	0.86	0.883	0.177	0.934
500	0.25	100	mrs	scumpbayes	0.86	0.845	0.905	0.343	0.933
500	0.5	100	mrs	scumpbayes	0.88	0.834	0.926	0.546	0.932
500	0.75	100	mrs	scumpbayes	0.913	0.815	0.946	0.756	0.927
500	0.95	100	mrs	scumpbayes	0.959	0.79	0.968	0.93	0.895
500	0.05	200	mrs	scumpbayes	0.936	0.949	0.701	0.084	0.956
500	0.25	200	mrs	scumpbayes	0.895	0.936	0.772	0.241	0.955
500	0.5	200	mrs	scumpbayes	0.873	0.926	0.819	0.447	0.954
500	0.75	200	mrs	scumpbayes	0.877	0.913	0.864	0.67	0.949
500	0.95	200	mrs	scumpbayes	0.909	0.894	0.909	0.869	0.917
500	0.05	400	mrs	scumpbayes	0.951	0.969	0.615	0.061	0.962
500	0.25	400	mrs	scumpbayes	0.894	0.959	0.699	0.205	0.961
500	0.5	400	mrs	scumpbayes	0.856	0.95	0.762	0.406	0.96
500	0.75	400	mrs	scumpbayes	0.847	0.94	0.816	0.627	0.956
500	0.95	400	mrs	scumpbayes	0.874	0.924	0.872	0.832	0.924

Table 9

Simulation study results for $n = 500$, non-uniform bots, and Supervised Classes, Unsupervised Mixing Proportions (SCUMP) Bayes classifier. Outcome measures are accuracy, specificity, sensitivity, flag rate, and area under the curve (AUC). n = target sample size, n; contam = contamination rate; n0tr = number of humans in calibration sample, n_0^{tr}; botdist = bot Likert response distribution; acc = accuracy; spec = specificity; sens = sensitivity; flag = flag rate; auc = area under the receiver operating characteristic curve; unif = uniform distribution; mrs = middle responding; scumpbayes = Bayes classifier with Supervised Classes, Unsupervised Mixing Proportions.
